# Assessment of the American Joint Commission on Cancer 8th Edition Staging System for Patients with Pancreatic Neuroendocrine Tumors: A Surveillance, Epidemiology, and End Results analysis

**DOI:** 10.1002/cam4.1336

**Published:** 2018-01-29

**Authors:** Xiaogang Li, Shanmiao Gou, Zhiqiang Liu, Zeng Ye, Chunyou Wang

**Affiliations:** ^1^ Department of Pancreatic Surgery Union Hospital Tongji Medical College Huazhong University of Science and Technology Wuhan 430022 China; ^2^ Department of General Surgery Xiangyang Central Hospital Affiliated Hospital Of Hubei University of Arts and Science Xiangyang 441021 China

**Keywords:** AJCC, ENETS, pancreatic neuroendocrine tumors, prognosis, staging system

## Abstract

Although several staging systems have been proposed for pancreatic neuroendocrine tumors (pNETs), the optimal staging system remains unclear. Here, we aimed to assess the application of the newly revised 8th edition American Joint Committee on Cancer (AJCC) staging system for exocrine pancreatic carcinoma (EPC) to pNETs, in comparison with that of other staging systems. We identified pNETs patients from the Surveillance, Epidemiology, and End Results (SEER) database (2004–2014). Overall survival was analyzed using Kaplan–Meier curves with the log‐rank test. The predictive accuracy of each staging system was assessed by the concordance index (c‐index). Cox proportional hazards regression was conducted to calculate the impact of different stages. In total, 2424 patients with pNETs, including 2350 who underwent resection, were identified using SEER data. Patients with different stages were evenly stratified based on the 8th edition AJCC staging system for EPC. Kaplan–Meier curves were well separated in all patients and patients with resection using the 8th edition AJCC staging system for EPC. Moreover, the hazard ratio increased with worsening disease stage. The c‐index of the 8th edition AJCC staging system for EPC was similar to that of the other systems. For pNETs patients, the 8th edition AJCC staging system for EPC exhibits good prognostic discrimination among different stages in both all patients and those with resection.

## Introduction

Pancreatic neuroendocrine tumors (pNETs), which account for 3–5% of all cases of pancreatic cancer, are uncommon malignancies, as compared with pancreatic adenocarcinoma 1, 2. The incidence of pNETs is 2.5–5 in 100,000 individuals per year worldwide and has rapidly increased due to improvements in imaging 3, 4, 5. Moreover, pNETs exhibit high heterogeneity in terms of tumor histology and clinical presentation. The tumor histology varies from well‐differentiated neoplasms, whose clinical behavior can be inactive or highly malignant, to poorly differentiated tumors, which generally exhibit poor prognosis 6. Based on the clinical presentation, pNETs can be classified into two types: functional tumors and nonfunctional tumors 7.

Due to the rarity and heterogeneity of pNETs, it is challenging to stratify patients into different survival risk groups. Of the various staging systems used for pNETs, the systems proposed by the Europe the European Neuroendocrine Tumor Society (ENETS) and the American Joint Committee on Cancer (AJCC) have been most widely used 8, 9, 10, 11. In 2006, ENETS introduced a TNM classification system for pNETs (Table 1). However, several studies found that patients with stage I disease had a similar prognosis to patients with stage II disease 12, 13. With regard to the AJCC staging system, it was initially only applied for exocrine pancreatic carcinoma (EPC). In 2007, Bilimoria et al. 14 applied the 6th edition AJCC staging system for patients with pNETs and showed that the staging system could effectively stratify patients with pNETs. In its 7th edition manual in 2010, the AJCC proposed the application of the EPC TNM‐staging system to pNETs, and this proposal was assessed in several other studies 6, 15, 16. Some studies showed that only a few patients were classified as stage III because pNETs seldom invade the celiac or mesenteric arteries 17. Due to the limitations of the ENETS and AJCC staging systems, studies have focused on the modifications of these staging systems. Luo et al. 10 developed a modified ENETS system (mENETS), which maintains the ENETS T, N, and M definitions and adopts the AJCC system staging definitions; the researchers found that the mENETS staging system was better than the ENETS and AJCC staging systems in stratifying patients with pNETs. In 2017, the AJCC incorporated several changes into the 8th edition system of EPC. The T and N definitions and other staging definitions were revised. Instead of being representative of extrapancreatic invasion, T2 and T3 tumors were now defined as those with a maximum tumor diameter of >2 cm, ≤4 cm, and >4 cm. Moreover, the N definition has been revised from a binary system to a tripartite system. With regard to the staging definitions, in addition to tumors with T4, any N, and M0, those with any N, T2, and M0 are also classified as stage III. Recently, the 8th edition AJCC edition staging system has been validated to enable the fine stratification of patients with pancreatic adenocarcinoma. However, the 8th Edition AJCC Cancer Staging Manual introduced a different staging system from that of EPC for pNETs, which is consistent with the ENETS staging system.

**Table 1:** Definitions of the four staging systems for pancreatic neuroendocrine tumors

	8th edition AJCC for EPC	7th edition AJCC	ENETS	mENETS
Primary tumor (T)				
T1	Maximum tumor diameter ≤2 cm	Tumor limited to the pancreas, ≤2 cm in its greatest dimension	Tumor limited to the pancreas, <2 cm	Tumor limited to the pancreas, <2 cm
T2	Maximum tumor diameter >2 cm but ≤4 cm	Tumor limited to the pancreas, >2 cm in its greatest dimension	Tumor limited to the pancreas, 2–4 cm	Tumor limited to the pancreas, 2–4 cm
T3	Maximum tumor diameter >4 cm	Tumor extends beyond the pancreas but without the involvement of the celiac axis or the superior mesenteric artery	Tumor limited to the pancreas, >4 cm, or invading the duodenum or common bile duct	Tumor limited to the pancreas, >4 cm, or invading the duodenum or common bile duct
T4	Tumor involves the celiac axis or the superior mesenteric artery (unresectable primary tumor)	Tumor involves the celiac axis or the superior mesenteric artery (unresectable primary tumor)	Tumor invades the adjacent structures	Tumor invades the adjacent structures
Lymph nodes (N)				
N0	No regional lymph node metastasis	No regional lymph node metastasis	No regional lymph node metastasis	No regional lymph node metastasis
N1	Metastasis in 1–3 regional lymph nodes	Regional lymph node metastasis	Regional lymph node metastasis	Regional lymph node metastasis
N2	Metastasis in ≥4 regional lymph nodes			
Metastases (M)				
M0	No distant metastasis	No distant metastasis	No distant metastasis	No distant metastasis
M1	Distant metastasis	Distant metastasis	Distant metastasis	Distant metastasis
Stage				
I	T1, N0, M0 (A)	T1, N0, M0 (A)	T1, N0, M0	T1, N0, M0 (A)
	T2, N0, M0 (B)	T2, N0, M0 (B)		T2, N0, M0 (B)
II	T3, N0, M0 (A)	T3, N0, M0 (A)	T2, N0, M0 (A)	T3, N0, M0 (A)
	T1‐3, N1, M0 (B)	T1‐3, N1, M0 (B)	T3, N0, M0 (B)	T1‐3, N1, M0 (B)
III	Any T, N2, M0	T4, any N, M0	T4, N0, M0 (A)	T4, any N, M0
	T4, any N, M0		Any T, N1, M0 (B)	
IV	Any T, any N, M1	Any T, any N, M1	Any T, any N, M1	Any T, any N, M

AJCC, American Joint Committee on Cancer; EPC, exocrine pancreatic carcinoma; ENETS, European Neuroendocrine Tumor Society; mENETS, modified European Neuroendocrine Tumor Society.

To verify whether the application of the 8th edition system for EPC is suitable for pNETs, we performed a population‐based study to assess four staging systems (8th edition AJCC for EPC, 7th edition AJCC, 8th edition AJCC for pNETs/ENETS, and mENETS) in patients with pNETs using the Surveillance, Epidemiology, and End Results (SEER) database.

## Materials and Methods

### Data source and patient data collection

This study was conducted using the SEER database, which is a well‐designed electronic medical record database for cancer research. Data on patient demographics, clinical tumor characteristics, the first course of treatment, and follow‐up for vital status were acquired from the SEER database using SEER*Stat software (version 8.3.4; National Cancer Institute, Bethesda, MD, USA). Patients with pNETs were identified using the ICD‐O‐3 histology codes (8150‐8153, 8155, 8156, 8240, 8241‐8243, 8246, and 8249). We assessed the 7th edition AJCC, 8th edition AJCC, ENETS, and mENETS staging systems using the following codes: CS tumor size (2004+), CS extension (2004+), CS lymph nodes (2004+), CS mets at dx (2004+), regional nodes positive (1988+).

From 2004 to 2014, a total of 6304 cases with pNETs were reviewed. The inclusion criteria were an age of ≥18 years old and a confirmed pathological diagnosis. Patients with incomplete information on the T, N, and M stages and the follow‐up period were excluded to reduce the selection bias. A total of 2424 patients were finally enrolled in this study. The survival duration was recorded from diagnosis to the date of death or last follow‐up. As no special personal information was recorded, ethical consent was not needed.

### Statistical analysis

Statistical analyses were conducted using STATA 14 software (StataCorp; College Station, TX). Continuous variables are presented as median and interquartile range, and categorical variables are expressed as frequency and percentage. Survival analyses were performed using the Kaplan–Meier model with the log‐rank test. A Harrell's concordance index (c‐index) was calculated for each staging system to assess its usefulness in correctly predicting patients at high or low risk of mortality 18, 19. The hazard ratios (HR) and 95% confidence intervals (95% CI) were computed using the Cox proportional hazards model. The variables selected for use in the multivariate analysis were based on findings in the previous literature and background knowledge. Statistical significance was defined as results with two‐tailed *P* values of < 0.05.

## Results

### Characteristics and clinical features of the entire cohort

In total, 2424 patients presenting with pNETs between 2004 and 2014 met the study criteria. The clinical characteristics of the study cohort are presented in Table 2. At baseline, the median age of the cohort was 59 years. Most of the patients were diagnosed between the ages of 49 and 68 years. Among the patients, 1299 (53.6%) were male and 1125 (46.4%) were female. The cohort comprised 79.9% Caucasian patients, 10.8% patients of African origin, and 8.6% patients of other races; a total of 2350 (97.0%) patients had undergone tumor resection. The 3‐year, 5‐year, and 10‐year survival rate was 84.7%, 74.6%, and 55.4%, respectively.

**Table 2:** Baseline characteristics of patients in the SEER database (*n* = 2424)

Characteristics	Number (%)
Age at diagnosis, median (IQR)	59 (49–68)
Sex	
Male	1299 (53.6)
Female	1125 (46.4)
Location	
Head	762 (31.4)
Body/Tail	1220 (50.3)
Other	442 (18.2)
Grade	
Low, intermediate	1863 (61.7)
High	157 (15.2)
Unknown	404 (16.7)
Year	
2004–2008	613 (25.3)
2009–2014	1811 (74.7)
Tumor type	
Nonfunctional	2367 (97.6)
Functional	57 (2.4)
Surgery	
Yes	2350 (97.0)
No	74 (3.0)
Marital status	
Married	1591 (65.6)
Single	410 (14.2)
Other	462 (15.5)
Unknown	113 (4.7)
Race	
Caucasian	1937 (79.9)
Of African origin	262 (10.8)
Other	208 (9.4)
Unknown	17 (0.7)

IQR, interquartile range; SEER database, the Surveillance, Epidemiology, and End Results database.

### Survival with the 8th edition AJCC staging system for EPC

**Figure 1:**
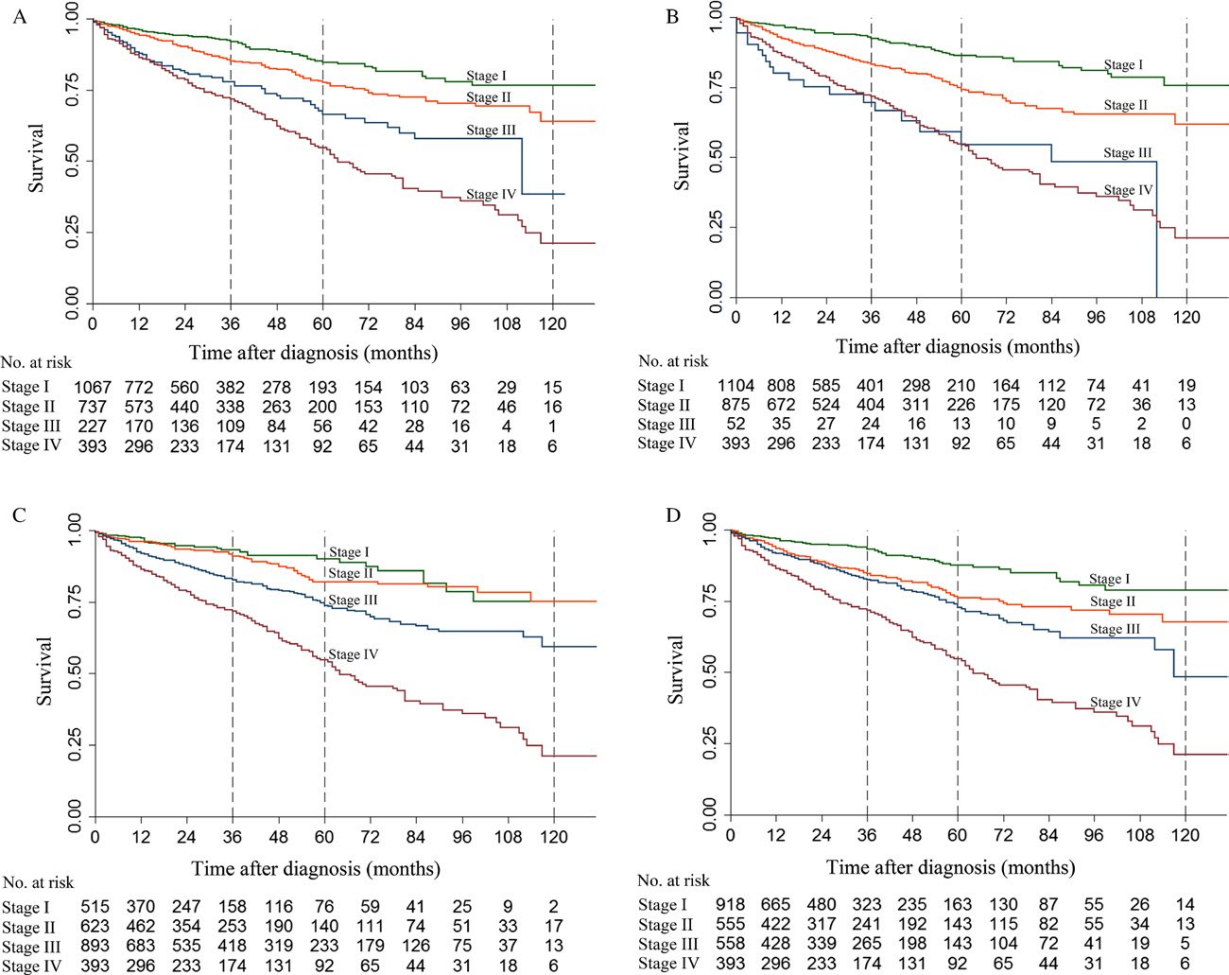
Kaplan–Meier survival curves of different staging systems for patients with pNETs from the SEER database in the whole population. (A) Kaplan–Meier survival curves for the 8th edition American Joint Committee on Cancer staging system (AJCC) for exocrine pancreatic carcinoma. (B) Kaplan–Meier survival curves for the 7th edition AJCC. (C) Kaplan–Meier survival curves for the 8th edition AJCC staging system for pNETs/the European Neuroendocrine Tumor Society (ENETS) staging system. (D). Kaplan–Meier survival curves for the modified ENETS staging system (mENETS).

With the 8th edition AJCC staging system for EPC, the survival differences among four stages were significant (*P *<* *0.05; Fig. 1A). The 3‐year survival rates for stages I, II, III, and IV were 91.9%, 85.1%, 77.8%, and 71.8%, respectively; the 5‐year survival rates were 84.6%, 77.6%, 66.3%, and 54.7%, respectively; and the 10‐year survival rates were 76.5%, 63.9%, 38.5%, and 21.2%, respectively (Table S1, Supporting information). In multivariable analyses, the HR significantly increased as the disease stage worsened (Table S2, supporting information). Moreover, the survival differences among the new T (*P *<* *0.05) and N statuses (*P *<* *0.05) were also significant (Fig. S1, Supporting information).

**Figure 2:**
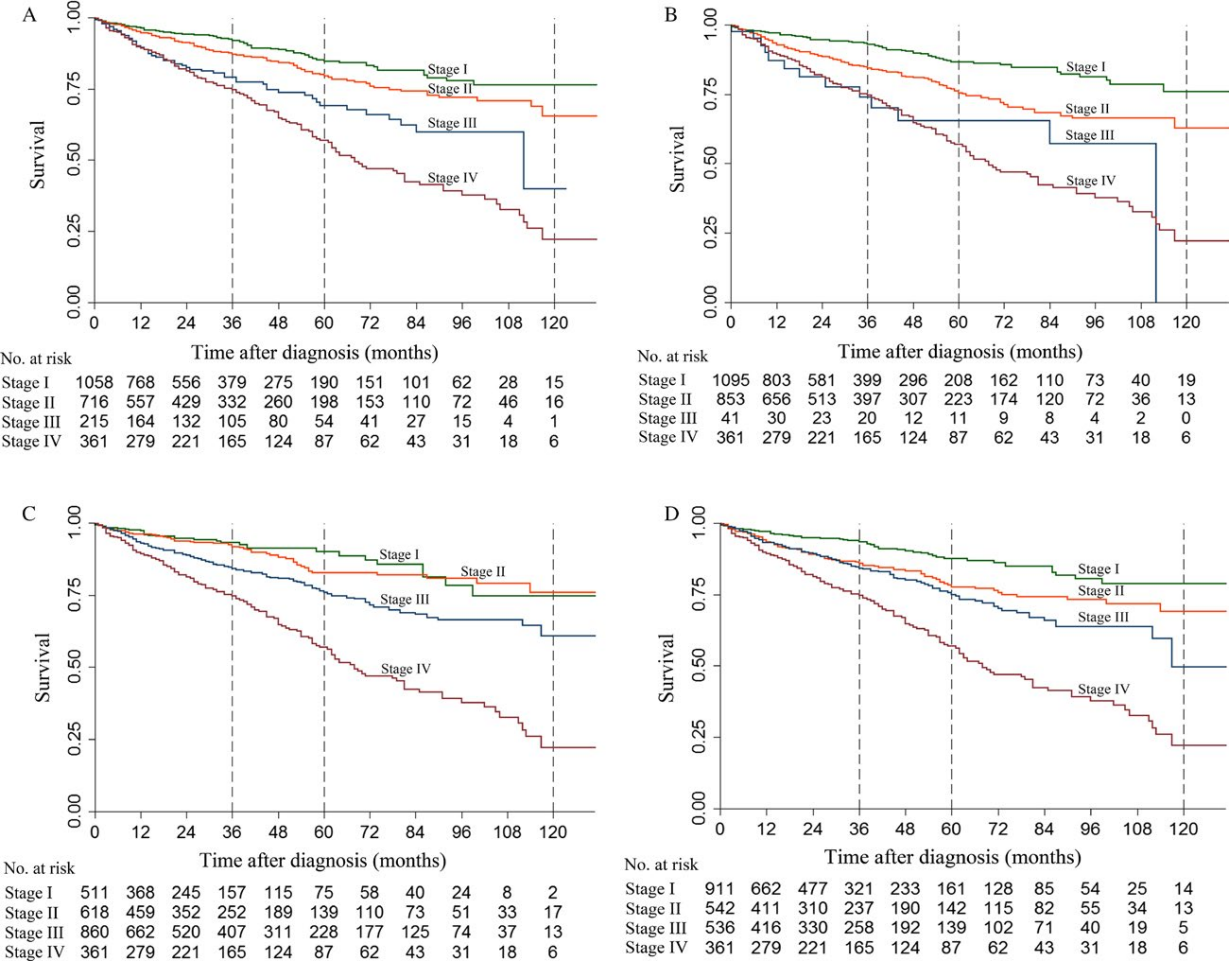
Kaplan–Meier survival curves of different staging systems for patients with pNETs from the SEER database in patients undergoing oncologic resection. (A) Kaplan–Meier survival curves for the 8th edition American Joint Committee on Cancer staging system (AJCC) for exocrine pancreatic carcinoma. (B) Kaplan–Meier survival curves for the 7th edition AJCC. (C) Kaplan–Meier survival curves for the 8th edition AJCC staging system for pNETs/the European Neuroendocrine Tumor Society (ENETS) staging system. (D) Kaplan–Meier survival curves for the modified ENETS staging system (mENETS).

In patients undergoing resection, the survival differences among each stage were also significant after 24 months (*P *<* *0.05; Fig. 2A). The 3‐year survival rates for stages I, II, III, and IV were 93.6%, 88.5%, 78.9%, and 74.7%, respectively; the 5‐year survival rates were 84.7%, 79.5%, 69.0%, and 56.9%, respectively; and the 10‐year survival rates were 76.4%, 65.5%, 39.9%, and 22.2%, respectively. The HR trends were similar to those noted in the whole population (Table S3, Supporting information).

### Survival with the 7th edition AJCC staging system

With the 7th edition AJCC staging system, only 52 (2.2%) patients were classified as stage III (Table 3). The Kaplan–Meier curves showed that patients with stage IV disease even experienced better survival than patients with stage III disease before 36 months and after 112 months, while the overall difference between the two stages was not significant (*P *=* *0.85; Fig. 1B). The 3‐year survival rates for stages I, II, III, and IV were 92.4%, 83.2%, 69.5%, and 71.8%, respectively; the 5‐year survival rates were 86.2%, 74.2%, 54.5%, and 54.7%, respectively; and the 10‐year survival rates were 75.7%, 61.8%, 0, and 21.2%, respectively. Multivariable analyses indicated that the HR increased as the stage worsened (Table S2, Supporting information).

**Table 3:** Distribution of the four staging systems in the study

Stage	8th edition AJCC for EPC *n* = 2424	7th edition AJCC *n* = 2424	ENETS *n* = 2424	mENETS *n* = 2424
All patients (*n* = 2424)				
I	1067 (44.0%)	1104 (45.5%)	515 (21.3%)	918 (37.9%)
II	737 (30.4%)	875 (36.1%)	623 (25.7%)	555 (22.9%)
III	227 (9.4%)	52 (2.2%)	893 (38.8%)	558 (23.0%)
IV	393 (16.2%)	393 (16.2%)	393 (16.2%)	393 (16.2%)
Patients who underwent resection (*n* = 2350)				
I	1058 (45.0%)	1095 (46.6%)	511 (21.7%)	911 (38.8%)
II	716 (30.5%)	853 (36.3%)	618 (26.3%)	542 (23.1%)
III	215 (9.2%)	41 (1.7%)	860 (36.6%)	536 (22.8%)
IV	361 (15.4%)	361 (15.4%)	361 (15.4%)	361 (15.3%)

AJCC, American Joint Committee on Cancer; EPC, exocrine pancreatic carcinoma; ENETS, European Neuroendocrine Tumor Society; mENETS, modified European Neuroendocrine Tumor Society.

In patients who underwent resection, the survival difference between stages III and IV was still not significant (*P *=* *0.92; Fig. 2b). The 3‐year survival rates for stages I, II, III, and IV were 92.8%, 84.4%, 73.9%, and 74.7%, respectively; the 5‐year survival rates were 86.6%, 75.4%, 65.3%, and 56.9%, respectively; and the 10‐year survival rates were 75.9%, 62.8%, 0, and 22.2%, respectively. The HR trends were similar to those of the whole population (Table S3, supporting information).

### Survival with the 8th edition AJCC staging system for pNETs/the ENETS staging system

The Kaplan–Meier curves of stages I and II indicated that the survival difference between the two stages was not significant in all patients (*P *=* *0.33; Fig. 1c) and in patients undergoing resection (*P *=* *0.47; Fig. 2c). In all the patients, the 3‐year survival rates for stages I, II, III, and IV were 93.1%, 91.1%, 82.7%, and 71.8%, respectively; the 5‐year survival rates were 90.0%, 82.6%, 73.8%, and 54.7%, respectively; and the 10‐year survival rates were 75.1%, 75.3%, 59.2%, and 21.2%, respectively.

In patients who underwent resection, the 3‐year survival rates for stages I, II, III, and IV were 93.0%, 91.8%, 84.2%, and 74.7%, respectively; the 5‐year survival rates were 89.9%, 82.6%, 75.8%, and 56.9%, respectively; and the 10‐year survival rates were 74.6%, 75.8%, 60.8%, and 22.2%, respectively. Multivariable analyses indicated that the death risk of stage II was not significant compared to stage I both in the whole population (HR, 1.32; 95% CI, 0.86–2.03) and in patients undergoing resection (HR, 1.25; 95% CI, 0.81–1.92).

### Survival with the mENETS staging system

With the modified ENETS staging system, the survival difference between stages II and III was not significant in the whole population (*P *=* *0.07; Fig. 1D) or in patients undergoing resection (*P *=* *0.14; Fig. 2D). In all the patients, the 3‐year survival rates for stages I, II, III, and IV were 93.2%, 84.6%, 82.4%, and 71.8%, respectively; the 5‐year survival rates were 87.4%, 76.1%, 72.8%, and 54.7%, respectively; and the 10‐year survival rates were 78.9%, 67.7%, 48.3%, and 21.2%, respectively. In patients who underwent resection, the 3‐year survival rates for stages I, II, III, and IV were 93.3%, 86.0%, 84.2%, and 74.7%, respectively; the 5‐year survival rates were 87.4%, 77.6%, 75.0%, and 56.9%, respectively; and the 10‐year survival rates were 78.7%, 69.0%, 49.6%, and 22.2%, respectively.

The HR of stage II was close to that of stage III (HRs of stages II and III in the whole population, 2.02 and 2.15, respectively; HRs of stages II and III in patients undergoing resection, 1.93 and 2.11, respectively).

### Comparison of predictive accuracy for overall survival among the four staging systems

As mentioned previously, the 8th edition AJCC staging system for EPC showed better prognostic stratification for patients as compared to the other three systems. The c‐indexes of the 8th edition AJCC for EPC, 7th edition AJCC, the 8th edition AJCC for pNETs/ENETS, and mENETS systems were 0.641 (95% CI, 0.613–0.669), 0.641 (95% CI, 0.613–0.669), 0.639 (95% CI, 0.612–0.666), and 0.642 (95% CI, 0.615–0.669), respectively; however, the differences were not significant (8th edition AJCC for EPC vs. 7th edition AJCC: *P *=* *0.304; 8th edition AJCC for EPC vs. the 8th edition AJCC for pNETs/ENETS: *P *=* *0.804; 8th edition AJCC vs. mENETS: *P *=* *0.943).

For patients undergoing resection, the c‐indexes of the 8th edition AJCC for EPC, 7th edition AJCC, the 8th edition AJCC for pNETs/ENETS, and mENETS systems were 0.627 (95% CI, 0.597–0.657), 0.627 (95% CI, 0.597–0.657), 0.625 (95% CI, 0.596–0.654), and 0.627 (95% CI, 0.598–0.657), respectively; similarly, the differences in the c‐indexes were not significant (8th edition AJCC for PADC vs. 7th edition AJCC: *P *=* *0.410; 8th edition AJCC for PADC vs. the 8th edition AJCC for pNETs/ENETS: *P *=* *0.831; 8th edition AJCC vs. mENETS: *P *=* *0.949).

## Discussion

No consensus has been reached regarding the staging system for pNETs. The ENETS staging system is widely used in Europe, and patients with pNETs were distributed evenly using this system. However, the present study and certain other reports suggest that ENETS lacks appropriate prognostic discrimination between stage I and stage II cases 6, 10, 12, 13. In 2007, Bilimoria et al. 14 applied the AJCC staging system of pancreatic adenocarcinoma to pNETs and found good prognostic discrimination between consecutive tumor stages. Based on this study, the 7th edition AJCC manual advised using the staging system of EPC for pNETs. However, due to the biologic differences between pancreatic adenocarcinoma and pNETs, the 7th staging AJCC system had certain limitations, including the stratification of a very low proportion of patients to stage III and poor prognostic discrimination between stage III and stage IV. However, given the salient features of the ENETS and 7th edition AJCC systems, Luo et al. 10 proposed a modified system for pNETs that combined both these systems; the modified staging system was considered to be better than the ENETS and AJCC staging systems in terms of stratifying patients with pNETs.

In 2016, the AJCC released the 8th edition manual with significant changes for EPC, including new definitions for T and N and the staging classification. Some studies validated the 8th edition AJCC staging system for EPC and found that the new system provided a better stratification of patients across different stages as compared to the AJCC 7th staging system 20, 21, 22. However, the 8th edition AJCC Cancer Staging Manual introduced a staging system very close to the ENETS system, in which the newly revised N category was not employed. Thus, we applied the 8th edition AJCC staging system for EPC to pNETs and compared this system with the other staging systems. Our results showed that the 8th edition AJCC staging system for EPC provided a finer prognostic discrimination than other staging systems, although the c‐index was similar to those of other systems.

The most criticized limitation of the 7th edition AJCC staging system was that only a few patients could be classified as stage III. Compared with the 7th edition AJCC staging system, the 8th edition AJCC staging system for EPC stratified more patients into stage III (from 2.2% to 9.4%), primarily due to the revision of stage III definition. Besides the retention of T4, any N, and M0, the stage III classification of the 8th edition AJCC staging system for EPC also included any T, N2, and M0 cases. The definitions of T and N were also revised in the 8th edition AJCC edition for EPC. The revision of stage, T and N definitions contribute to the better stratification of patients with pNETs, as compared to other staging systems. As shown in Figure S1a, those with stages N1 and N2 (who were all with stage N1 in 7th edition AJCC system) showed significantly different prognosis. Thus, the modification of the N‐stage definition theoretically should markedly contribute to the stratification of patients. With regard to the modification of the T‐stage definition, it is interesting to note that the T stage of the 7th edition AJCC system appears to be better than that of the 8th edition AJCC system for EPC in terms of stratifying those with different stages (Fig S1b and S1c). When the T‐stage definition of the 7th edition AJCC system was used for the 8th edition AJCC system for EPC (mAJCC8), the new staging system showed greater c‐index values in the whole patient population (0.650; 95% CI, 0.623–0.677) and in patients undergoing oncologic surgery (0.639; 95% CI, 0.610–0.668). Nevertheless, no significant improvement was observed when stratifying patients with pNETs using the mAJCC8 staging system (Fig. S2). In addition, the exclusion of tumor extension in the T definition of the 8th edition AJCC system for EPC may make the staging system more practical because the pancreas lacks a true capsule and because the evaluation of peripancreatic soft tissue involvement may be difficult due to the desmoplastic reaction between the pancreas and the peripancreatic soft tissue 23.

The present study has several limitations due to the use of SEER data. First, some important prognostic factors were not recorded in the SEER database, such as margin status, chemotherapy, and comorbidity score. However, the effect of margin status may be limited, as the results of our study and Kamarajah et al. 20 were similar to those of Allen et al. 21 who used the R0 cohort. Second, the SEER data lacked a centralized pathological review. However, Field et al. 24 found a fine coincidence between the SEER histological subtypes and those assessed by independent reviewers. Third, some patients with an unknown number of positive lymph nodes were excluded from our study. This selection bias might limit the generalizability of our study. Despite these limitations, it was reasonable to use the SEER database due to the rarity and heterogeneity of pNETs.

In conclusion, our study validated that the 8th edition AJCC staging system for EPC is also suitable for pNETs using a population‐based database. Our results show that the 8th edition AJCC staging system for EPC demonstrates good prognostic discrimination between the different stages for both the whole population of patients with pNETs and patients undergoing oncological resection. Besides, it must be pointed out that pNETs are an entity with high heterogeneity on biologic behavior and prognosis. According to the classification of the WHO, pNETs were subclassified into subgroups, that is G1, G2, and G3, based on Ki‐67 index and mitosis, and different groups showed different prognosis 25. Recently, the G3 pNETs were suggested to be further subclassified into G3 NET and G3 NEC 26. Different from EPC, the heterogeneity of pNETs among groups was so great that different treatment strategies were suggested to different subgroups in practical guidelines 26, 27, 28, while the treatment strategies for EPC were consistent. Thus, combination of TNM‐staging system and tumor grade of pNETs should improve the prediction of the prognosis, but how to quantify the combination is still to be further studied 29, 30.

## Conflict of Interest

None declared.

## Supporting information


**Table S1**. Survival rate of cases with different staging systems.
**Table S2**. Multivariate analysis of the prognostic factors from the SEER database in the whole population.
**Table S3**. Multivariate analysis of the prognostic factors from the SEER database in patients who underwent resection.
**Figure S1**. Kaplan–Meier survival curves of different N status for patients with pNETs according to the 8th edition American Joint Committee on Cancer staging system for exocrine pancreatic carcinoma (a). Kaplan–Meier survival curves of different T status for patients with pNETs according to the 8th edition American Joint Committee on Cancer staging system for exocrine pancreatic carcinoma (b) and the 7th edition American Joint Committee on Cancer staging system (c).
**Figure S2**. Kaplan–Meier survival curves of the modified 8th edition American Joint Committee on Cancer staging system for patients with pNETs in the whole population (a). Kaplan–Meier survival curves of the modified 8th edition American Joint Committee on Cancer staging system for patients with pNETs in patients undergoing oncologic resection (b).
