# Supplementary material for: Assessment of the American Joint Commission on Cancer 8th Edition Staging System for Patients with Pancreatic Neuroendocrine Tumors: A Surveillance, Epidemiology, and End Results analysis
Source: Cancer Med. 2018 Jan 29;7(3):626–34. doi: 10.1002/cam4.1336 (PMC5852336; doi:10.1002/cam4.1336)
Supplement: Supplementary file 1 — Table S1. Survival rate of cases with different staging systems. Table S2. Multivariate analysis of the prognostic factors from the SEER database in the whole population. Table S3. Multivariate analysis of the prognostic factors from the SEER database in patients who underwent resection. Figure S1. Kaplan–Meier survival curves of different N status for patients with pNETs according to the 8th edition American Joint Committee on Cancer staging system for exocrine pancreatic carcinoma (a). Kaplan–Meier survival curves of different T status for patients with pNETs according to the 8th edition American Joint Committee on Cancer staging system for exocrine pancreatic carcinoma (b) and the 7th edition American Joint Committee on Cancer staging system (c). Figure S2. Kaplan–Meier survival curves of the modified 8th edition American Joint Committee on Cancer staging system for patients with pNETs in the whole population (a). Kaplan–Meier survival curves of the modified 8th edition American Joint Committee on Cancer staging system for patients with pNETs in patients undergoing oncologic resection (b). [file CAM4-7-626-s001.pdf]

Assessment of the American Joint Commission on Cancer 8th Edition Staging System for Patients with Pancreatic Neuroendocrine Tumors: A Surveillance, Epidemiology and End Results Analysis

**Table S1: Survival rate of cases with different staging systems.**

| | | 3-year survival rate (%) | 5-year survival rate (%) | 10-year survival rate (%) |
|---|---|---|---|---|
| **The whole population** | | | | |
| **AJCC 8th for EPC** | Stage I | 91.9 (90.9-92.9) | 84.6 (82.8-86.4) | 76.5 (73.6-79.4) |
| | Stage II | 85.1 (83.6-86.6) | 77.6 (75.1-79.3) | 63.9 (59.5-68.3) |
| | Stage III | 77.8 (74.9-80.7) | 66.3 (62.3-70.3) | 38.5 (22.7-54.3) |
| | Stage IV | 71.8 (69.4-74.3) | 54.7 (51.6-57.8) | 21.2 (16.1-26.3) |
| **AJCC 7th** | Stage I | 92.4 (91.9-93.9) | 86.2 (84.6-88.8) | 75.7 (71.9-79.5) |
| | Stage II | 83.2 (81.7-84.7) | 74.2 (72.2-76.2) | 61.8 (57.6-66.0) |
| | Stage III | 69.5 (62.6-76.4) | 54.5 (45.9-63.0) | 0 |
| | Stage IV | 71.8 (69.4-74.3) | 54.7 (51.6-57.8) | 21.2 (16.1-26.3) |
| **ENETS** | Stage I | 93.1 (91.7-94.4) | 90.0 (87.9-92.1) | 75.1 (69.4-80.8) |
| | Stage II | 91.1 (89.7-92.5) | 82.6 (80.3-84.9) | 75.3 (71.1-79.5) |
| | Stage III | 82.7 (81.2-84.2) | 73.8 (71.8-75.8) | 59.2 (54.8-63.6) |
| | Stage IV | 71.8 (69.4-74.3) | 54.7 (51.6-57.8) | 21.2 (16.1-26.3) |
| **mENETS** | Stage I | 93.2 (92.2-94.2) | 87.4 (85.6-89.2) | 78.9 (75.7-82.1) |
| | Stage II | 84.6 (82.8-86.4) | 76.1 (73.7-78.6) | 67.7 (63.8-71.6) |
| | Stage III | 82.4 (80.5-84.3) | 72.8 (70.4-75.3) | 48.3 (38.7-57.9) |
| | Stage IV | 71.8 (69.4-74.3) | 54.7 (51.6-57.8) | 21.2 (16.1-26.3) |
| **Patients who underwent resection** | | | | |
| **AJCC 8th for EPC** | Stage I | 93.6 (92.7-94.5) | 84.7 (82.9-86.5) | 76.4 (73.5-79.3) |
| | Stage II | 88.5 (87.1-89.9) | 79.5 (77.5-81.5) | 65.5 (61.0-70.0) |
| | Stage III | 78.9 (75.9-81.9) | 69.0 (65.1-72.9) | 39.9 (23.5-56.3) |
| | Stage IV | 74.7 (72.1-77.2) | 56.9 (53.6-60.2) | 22.2 (16.8-27.6) |
| **AJCC 7th** | Stage I | 92.8 (91.8-93.8) | 86.6 (85.0-88.2) | 75.9 (72.1-79.7) |
| | Stage II | 84.4 (83.0-85.8) | 75.4 (73.4-77.4) | 62.8 (58.5-67.1) |
| | Stage III | 73.9 (66.4-81.4) | 65.3 (56.6-74.0) | 0 |
| | Stage IV | 74.7 (72.2-77.2) | 56.9 (53.6-60.2) | 22.2 (16.8-27.6) |
| **ENETS** | Stage I | 93.0 (91.6-94.4) | 89.9 (87.8-92.0) | 74.6 (68.7-80.5) |
| | Stage II | 91.8 (90.5-93.1) | 82.6 (80.3-84.9) | 75.8 (71.6-80.0) |
| | Stage III | 84.2 (82.8-85.6) | 75.8 (73.8-77.8) | 60.8 (56.3-65.3) |
| | Stage IV | 74.7 (72.2-77.2) | 56.9 (53.6-60.2) | 22.2 (16.8-27.6) |
| **mENETS** | Stage I | 93.3 (92.3-94.3) | 87.4 (85.6-89.2) | 78.7 (75.4-82.0) |
| | Stage II | 86.0 (84.3-87.7) | 77.6 (75.2-80.1) | 69.0 (65.0-73.0) |
| | Stage III | 84.2 (82.4-86.0) | 75.0 (72.6-77.5) | 49.6 (39.7-59.5) |
| | Stage IV | 74.7 (72.2-77.2) | 56.9 (53.6-60.2) | 22.2 (16.8-27.6) |

Abbreviations: AJCC, American Joint Committee on Cancer; EPC, exocrine pancreatic carcinoma; ENETS, European Neuroendocrine Tumor Society; mENETS, modified European Neuroendocrine Tumor Society.

**Table S2**: **Multivariate analysis of the prognostic factors from the SEER database in the whole population.**

| Variable | 8th edition AJCC for EPC HR (95% CI) | *P*-value | 7th edition AJCC HR (95% CI) | *P*-value | ENETS HR (95% CI) | *P*-value | mENETS HR (95% CI) | *P*-value |
|---|---|---|---|---|---|---|---|---|
| **Age** | 1.03 (1.02, 1.04) | <0.001 | 1.03 (1.02, 1.04) | <0.001 | 1.03 (1.02, 1.04) | <0.001 | 1.03 (1.02, 1.04) | <0.001 |
| **Sex** | | | | | | | | |
| Female *vs* Male | 0.75 (0.61, 0.91) | 0.004 | 0.75 (0.62, 0.91) | 0.004 | 0.75 (0.61, 0.91) | 0.003 | 0.75 (0.62, 0.92) | 0.005 |
| **Location** | | | | | | | | |
| Head | Reference | | Reference | | | | | |
| Body/tail | 0.72 (0.58, 0.90) | 0.004 | 0.74 (0.59, 0.93) | 0.009 | 0.71 (0.58, 0.89) | 0.001 | 0.72 (0.57, 0.90) | 0.004 |
| Other | 0.98 (0.76, 1.28) | 0.905 | 1.00 (0.77, 1.30) | 0.997 | 0.97 (0.75, 1.27) | 0.836 | 0.98 (0.75, 1.27) | 0.864 |
| **Grade** | | | | | | | | |
| Low, intermediate | Reference | | Reference | | | | | |
| High | 3.26 (2.49, 4.27) | <0.001 | 3.14 (2.39, 4.11) | <0.001 | 3.26 (2.49, 4.27) | <0.001 | 3.26 (2.49, 4.27) | <0.001 |
| Unknown | 1.33 (1.05, 1.68) | <0.001 | 1.35 (1.07, 1.71) | 0.013 | 1.34 (1.06, 1.70) | 0.014 | 1.34 (1.06, 1.70) | 0.015 |
| **Tumor type** | | | | | | | | |
| Nonfunctional | Reference | | Reference | | Reference | | Reference | |
| Functional | 1.14 (0.62, 2.09) | 0.671 | 1.35 (0.79, 2.32) | 0.143 | 1.36 (0.79, 2.34) | 0.260 | 1.36 (0.79, 2.33) | 0.266 |
| **Surgery** | | | | | | | | |
| Yes *vs* No | 0.52 (0.36, 0.75) | <0.001 | 0.54 (0.37, 0.80) | | 0.52 (0.36, 0.75) | <0.001 | 0.51 (0.35, 0.74) | <0.001 |
| **Stage** | | | | | | | | |
| I | Reference | | Reference | | Reference | | Reference | |
| II | 1.53 (1.15, 2.02) | 0.003 | 2.00 (1.52, 2.62) | <0.001 | 1.32 (0.86, 2.03) | 0.204 | 2.02 (1.46, 2.81) | <0.001 |
| III | 2.25 (1.60, 3.17) | <0.001 | 3.09 (1.78, 5.35) | <0.001 | 2.22 (1.52, 3.26) | <0.001 | 2.15 (1.57,3.00) | <0.001 |
| IV | 4.05 (3.09, 5.31) | <0.001 | 4.59 (3.49, 6.04) | <0.001 | 5.02 (3.41, 7.40) | <0.001 | 4.90 (3.62, 6.63) | <0.001 |

Abbreviations: AJCC, American Joint Committee on Cancer; EPC, exocrine pancreatic carcinoma; ENETS, European Neuroendocrine Tumor Society; mENETS, modified European Neuroendocrine Tumor Society; HR, hazard ratio; CI, confidence intervals.

**Table S3**: **Multivariate analysis of the prognostic factors from the SEER database in patients who underwent resection.**

| **Variable** | **8th edition AJCC for EPC HR (95% CI)** | ***P*-value** | **7th edition AJCC HR (95% CI)** | ***P*-value** | **ENETS HR (95%CI)** | ***P*-value** | **mENETS HR (95%CI)** | ***P*-value** |
|---|---|---|---|---|---|---|---|---|
| **Age** | 1.03 (1.02, 1.04) | <0.001 | 1.03 (1.02, 1.04) | <0.001 | 1.03 (1.02, 1.04) | <0.001 | 1.03 (1.02, 1.04) | <0.001 |
| **Sex** | | | | | | | | |
| Female *v* Male | 0.72 (0.59, 0.88) | 0.002 | 0.72 (0.58, 0.88) | 0.002 | 0.72 (0.58, 0.88) | 0.002 | 0.73 (0.62, 0.92) | 0.002 |
| **Location** | | | | | | | | |
| Head | Reference | | Reference | | | | | |
| Body/Tail | 0.70 (0.55, 0.89) | 0.002 | 0.72 (0.57, 0.91) | 0.009 | 0.68 (0.54, 0.86) | 0.001 | 0.69 (0.54, 0.86) | 0.001 |
| Other | 0.96 (0.72, 1.26) | 0.750 | 0.97 (0.74, 1.28) | 0.839 | 0.93 (0.71, 1.23) | 0.617 | 0.93 (0.71, 1.24) | 0.633 |
| **Grade** | | | | | | | | |
| Low, intermediate | Reference | | Reference | | | | | |
| High | 3.21 (2.42, 4.25) | <0.001 | 3.08 (2.33, 4.09) | <0.001 | 3.17 (2.40, 4.20) | <0.001 | 3.17 (2.39, 4.20) | <0.001 |
| Unknown | 1.33 (1.04, 1.70) | 0.021 | 1.36 (1.07, 1.74) | 0.013 | 1.36 (1.06, 1.73) | 0.014 | 1.35 (1.06, 1.73) | 0.015 |
| **Tumor type** | | | | | | | | |
| Nonfunctional | Reference | | Reference | | Reference | | Reference | |
| Functional | 1.26 (0.72, 2.20) | 0.415 | 1.29 (0.74, 2.25) | 0.377 | 1.30 (0.74, 2.26) | 0.374 | 1.29 (0.74, 2.26) | 0.371 |
| **Stage** | | | | | | | | |
| I | Reference | | Reference | | Reference | | Reference | |
| II | 1.45 (1.09, 1.93) | 0.012 | 1.99 (1.51, 2.62) | <0.001 | 1.25 (0.81, 1.92) | 0.204 | 1.93 (1.38, 2.70) | <0.001 |
| III | 2.30 (1.63, 3.26) | <0.001 | 3.60 (1.94, 6.68) | <0.001 | 2.13 (1.45, 3.14) | <0.001 | 2.11 (1.53, 2.92) | <0.001 |
| IV | 4.19 (3.18, 5.52) | <0.001 | 4.78 (3.61, 6.34) | <0.001 | 5.07 (3.43, 7.50) | <0.001 | 5.04 (3.71, 686) | <0.001 |

Abbreviations: AJCC, American Joint Committee on Cancer; EPC, exocrine pancreatic carcinoma; ENETS, European Neuroendocrine Tumor Society; mENETS, modified European Neuroendocrine Tumor Society; HR, hazard ratio; CI, confidence intervals.

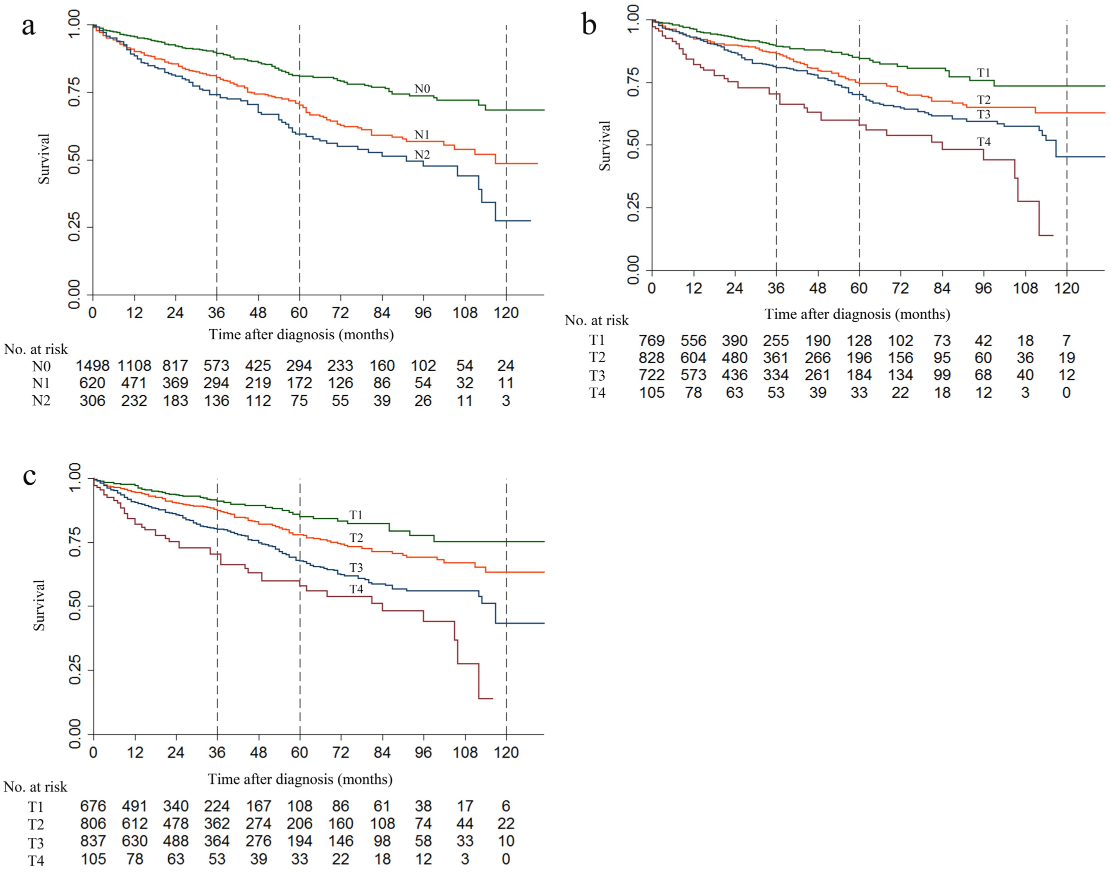


**Figure S1** Kaplan-Meier survival curves of different N status for patients with pNETs according to the 8th edition American Joint Committee on Cancer staging system for exocrine pancreatic carcinoma (**a**). Kaplan-Meier survival curves of different T status for patients with pNETs according to the 8th edition American Joint Committee on Cancer staging system for exocrine pancreatic carcinoma (**b**) and the 7th edition American Joint Committee on Cancer staging system (**c**).

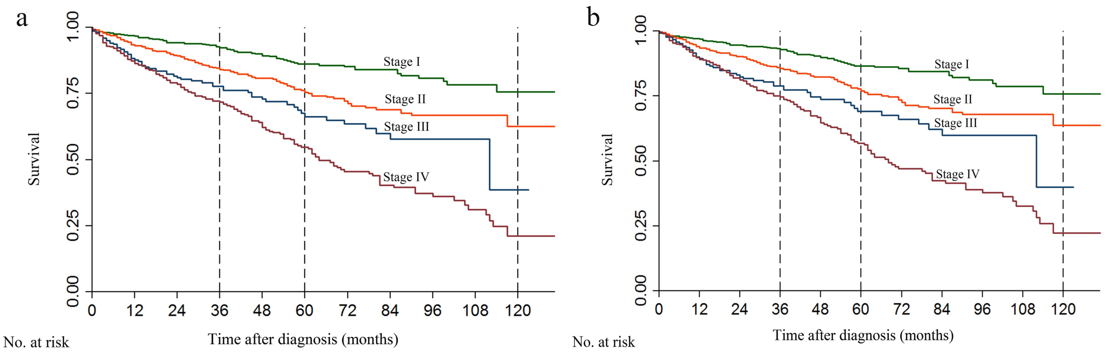


a

| No. at risk | | | | | | | | | | | |
|---|---|---|---|---|---|---|---|---|---|---|---|
| Stage I | 1105 | 808 | 585 | 401 | 298 | 210 | 164 | 112 | 74 | 41 | 19 |
| Stage II | 699 | 537 | 415 | 319 | 243 | 183 | 143 | 101 | 61 | 34 | 12 |
| Stage III | 227 | 170 | 136 | 109 | 84 | 56 | 42 | 28 | 16 | 4 | 1 |
| Stage IV | 393 | 296 | 233 | 174 | 131 | 92 | 65 | 44 | 31 | 18 | 6 |

b

| No. at risk | | | | | | | | | | | |
|---|---|---|---|---|---|---|---|---|---|---|---|
| Stage I | 1095 | 803 | 581 | 399 | 296 | 208 | 162 | 110 | 73 | 40 | 19 |
| Stage II | 679 | 522 | 404 | 312 | 239 | 180 | 142 | 101 | 61 | 34 | 12 |
| Stage III | 215 | 164 | 132 | 105 | 80 | 54 | 41 | 27 | 15 | 4 | 1 |
| Stage IV | 361 | 279 | 221 | 165 | 124 | 87 | 62 | 43 | 31 | 18 | 6 |

**Figure S2** Kaplan-Meier survival curves of the modified 8th edition American Joint Committee on Cancer staging system for patients with pNETs in the whole population (**a**). Kaplan-Meier survival curves of the modified 8th edition American Joint Committee on Cancer staging system for patients with pNETs in patients undergoing oncologic resection (**b**). In the modified 8th edition American Joint Committee on Cancer staging system, the T stage definition of the 7th edition American Joint Committee on Cancer staging system was used.
